# A Letter to the Commissioners for Transports, Sick and Wounded Seamen, &c. on the Subject of the Operation for Popliteal Aneurism: Illustrated by Cases, and the Description of a New Instrument

**Published:** 1818-03

**Authors:** 


### PART II.

### COMPREHENSIVE ANALYTICAL REVIEW OF MEDICAL LITERATURE.

"Tros, tyriusve, nobis nullo discrimine agetur."

*A Letter to the Commissioners for Transports, Sick and Wounded Seamen, &c. on the Subject of the Operation for Popliteal Aneurism: Illustrated by Cases, and the Description of a new Instrument.* By Alexander Copland Hutchison, M. D. (lately) Surgeon to the Royal Naval Hospital at Deal. pp. 19. London.

Dr. Hutchison is a man of diligence and intelligence, and is distinguished amongst his compeers by his great zeal for the promotion of chirurgical knowledge and improvement; — a zeal highly honourable and estimable when it is "according to knowledge." In addition to all this, his practical opportunities have not been inconsiderable. We, therefore, receive with respect any observations that are the offspring of his pen; and, on the present occasion, are willing to condense the useful facts which this little Tract contains, and give them "a local habitation" in our Analytical Repository.

It is written with the sole object of recommending the femoral artery, in cases of popliteal aneurism, to be taken up and tied, on the outside of the Sartorius muscle, nigh where the vessel penetrates the triceps, instead of on the inside, as pointed out and practised by Mr. John Hunter. There are also introduced the plate and description of a finger instrument, which the author has found greatly useful in assisting him to secure the artery in operations of this nature.

We shall present to our readers the objections to Mr. Hunter's mode of operating, in the writer's own words:

"By making the incision upon the inner margin of the Sartorius, the vena saphena major comes immediately in the way, and will be divided five times out of seven; consequently, a considerable flow of blood may be expected to ensue: but it is not the simple division of the vein, nor the probability of cutting off this channel for the returning blood of the leg, which are the reasons why this vessel should be avoided, for we all know the femoral vein itself to be fully adequate to the office; but it is the embarrassment which the bleeding will occasion to the operator, during the subsequent steps of so nice and intricate a dissection.

"In this direction, also, the principal lymphatics of the leg pass, which, most probably, will be divided with the vein; and should there exist much œdema of the leg (by no means an unusual circumstance where the aneurismal tumour happens to be large), the absorption of the effused serum will be tedious in the extreme.

"It frequently happens, likewise, that the external wound is kept open, for a considerable length of time after the ligatures have come away, by a profuse discharge of lymph from the wounded ends of these vessels." P. 3.

So much for the objections: — now let us turn to Dr. Hutchison's proposed improvement.

"About an inch, or an inch and half, above the part where the artery penetrates the tendon of the triceps muscle, is the point at which I would recommend it to be tied. There the Sartorius crosses the artery covered only by its sheath, and there the latter will be found fully as near the outer as the inner margin of the muscle. This being a clearly established fact, I would therefore ask, why should a preference be given to the method pursued by Mr. Hunter? for, surely, the lower the artery is tied on the fore part of the thigh for popliteal aneurism, the greater will be the chance of the inferior parts of the limb being properly nourished; and should any accident occur, such as secondary hæmorrhage, the artery may be tied higher up, or amputation may be performed with a much greater prospect of success, than if the vessel in the first instance were tied half way between the middle of the thigh and Poupart's ligament." P. 6.

Now, to all this we would reply, that we have performed the operation, and have always seen it performed, after the manner of the illustrious Hunter; and never once was the saphena vein divided, nor yet the principal lymphatic trunk of the thigh. The only difficulty, in general, experienced, is to detach the artery from the *fascia propria* which encloses the principal artery, vein, nerve, and lymphatic of the limb: this is a part of the operation requiring nice and cautious dissection, and the occasional use of the handle of the scalpel.

We would also allege, that the artery is at least *as* easily got at, in the usual place, inside the Sartorius muscle, as where it perforates the triceps:—therefore, why change, since even Dr. H. himself does not go the length of saying that it is *more* accessible in the latter situation?

When gangrene, from defective nutrition, or secondary hæmorrhage, renders amputation necessary, we cannot see how two or three inches of stump, more or less, can be of any consequence; or how a consideration of this nature can be of any avail in establishing the preferableness of one mode of operation for aneurism to another.

From the point where the femoral trunk gives off the profunda, till where it pierces the triceps, it gives off no branches — at least none, save capillary twigs to the adjacent cellular texture. How, then, can this new operation add to the chances of the limb being nourished?

Again — does it diminish the danger of secondary hæmorrhage, as Dr. H. seems to insinuate? We answer, No, quite the contrary; for it has been ascertained, over and over again, that in aneurisms, of any size or standing, there is always, we shall not say a *certainty*, but a *great risk*, of the arterial coats being diseased for a considerable way up the canal, above the aneurismal tumour. It was, indeed, to avoid this, amongst other evils, that Mr. Hunter put in practice his improved operation; an improvement which we deem one of the proudest on surgical record.

If we operate early, in cases of popliteal aneurism (as Mr. Ramsden and Dr. H. seem to advise), we forfeit, in the first place, the chance of a spontaneous cure; and, in the second place, we lessen the chances of the limb being adequately nourished, by having recourse to the knife ere the anastomosing branches have had time to dilate themselves. It is owing to this gradual and progressive dilatation, that the operation is so much more successful when performed for an aneurism, than for a wound of the popliteal artery. If we wait the requisite time, and at last tie the artery where it perforates the triceps, we shall probably have to encounter a diseased vessel, and many of the ill consequences which were wont too frequently to attend operations of this kind, before Mr. Hunter's time.'

Upon the whole, then, we are disposed to adhere to the old precept (laid down, we believe, by Mr. John Bell), of making our incision on an imaginary line drawn from the anterior superior spinous process of the ilium, to the inner condyle of the knee: suppose this line divided into five parts, the artery, we think, ought to be tied at the distance of three parts from the former, and two from the latter place; that is, about midway on the thigh, *inside* the Sartorius.

We have no decided opinion to give for or against the finger instrument which Dr. H. has here recommended. We must, however, remark generally, that the necessity for such contrivances will, in almost every instance, be superseded, if the external wound is made with becoming freedom. We have more than once had occasion to see operations for lithotomy, hernia, &c. productive of some embarrassment, even to a skilfnl operator, simply from his neglecting to make his first incisions with the necessary boldness, and of the necessary extent.

After venturing to differ thus freely from Dr. Hutchison, it is incumbent upon us to point out, with equal readiness, that wherein he has our hearty concurrence. We shall, therefore, transcribe the following remarks, which are very opposite to this, as well as to all other operations.

"In order to obviate the chance of large collections of matter forming, the first circumstance to which our attention will naturally be directed, is the speedy removal of the exciting causes of inflammation; and the chief of these will no doubt appear to be the ligatures, which, acting as extraneous bodies, keep up that degree of irritation in the wound, highly favourable to this secretion. It becomes, therefore, an object of no inconsiderable degree of interest, to accelerate the separation of these exciting causes, at as early a period as can be done with safety. With this view, it is my uniform practice, after the 12th day, in all capital operations where an artery of magnitude is concerned, to take hold of both ends of the ligature, to keep it gently on the stretch, and to twist it between the finger and thumb, which has the effect of tightening the noose. The ligature is then placed upon the adjoining sound parts, and retained in this twisted state, by means of a slip of adhesive plaster laid over it, until next dressing; when, should the ligature be found not detached from the end of the artery, the noose is tightened still more by the same process. In this way I have invariably succeeded, excepting in one instance, in removing the ligature after the 2d day of practising this simple operation, and that without a single drop of arterial blood following.

"His Majesty's ship Acasta arrived in the Downs from the West Indies, in August 1809; and, among a number of invalids sent from that ship to the hospital, was a black seaman, who had his arm amputated above the elbow six months previous to his admission: and, even at that distant period from the operation, there still remained a ligature hanging out from the stump, which, otherwise, was completely healed. Frequent attempts were made by the surgeon, during the passage home, to detach it, but without success. After the patient's admission into the hospital, I used every effort consistent with prudence, to remove it, but it was found so firmly attached, that I am persuaded the cord would have broken, rather than have separated at its noose. Both ends of this cord happened fortunately to be left together; and, after the second day of performing the operation of twisting, it came away, accompanied by a small, opake, hard, and irregularly round substance, not unlike a middle-sized pearl;—very probably the end of a nerve which had been included in the noose with the artery." P. 7.

We know not whether the manipulation here described would be equally successful, when one end of the ligature is cut away, which it now very generally is, the moment the arteries are tied.

At page 16, there is another practical remark, the justness of which, we presume, no one of the profession, at this time of day, will question. We quote it, in order to impress the fact more strongly on the minds of our younger readers.

"I conceive a secondary hæmorrhage, after this operation, is the grand point to be guarded against; and when it does occur, it is, in nine cases out of ten, owing to ulceration of the coats of the artery, from having its cellular connections to the surrounding parts destroyed above the ligature, which deprives the denuded vessel of its usual supply of nourishment."

From the importance of the subject, we have bestowed a degree of attention upon this little tract altogether disproportionate to its size. It certainly does no discredit to Dr. H.; yet our readers will easily collect, from what we have said, that we regard his proposal as an innovation without an improvement. Nevertheless, we thank him for the practical facts which it contains, and for the note at page 2d, where he vindicates the claims of English surgery, and the just fame of Mr. Hunter, from the ambitious and appropriating spirit of our French rivals, who, in the face of direct evidence, pertinaciously and unjustly ascribe to their own Anel, Guillemeau, and Dessault, the merit of this great Chirurgical improvement.

